# Mirai Chatterjee: community action for women’s health equity

**DOI:** 10.2471/BLT.24.030224

**Published:** 2024-02-01

## Abstract

Mirai Chatterjee talks to Fid Thompson about overcoming inequity and tackling the social determinants of health impacting female informal sector workers through collective action.

*Q: When did you develop your particular interest in informal sector women’s rights?*

A: I grew up aware of women’s place in the world of work because of my mother. My father, a promising nuclear physicist, died of cancer at the age of 28, which left my college-educated mother to raise me and my sister while also having to make a living. This was unusual in India 60 years ago, and I grew up hearing stories from her and her friends about how hard it was to be a working woman even in a cosmopolitan, relatively modern city like Mumbai. But I also had some exposure to low-income families through my school, and every summer my mother would take us to different places to open our eyes to the reality of poverty in India. So, by the time I got to university, the issue of poverty, inequality and injustice was very much on my mind.

*Q: When did you first learn about the Self-Employed Women's Association (SEWA)?*

A: When I was a student at Harvard, someone told me about its founder, Ela Bhatt – Elaben as she is affectionately known. Elaben had just won the prestigious Raymond Magsaysay Award for her work, and I found her very inspiring. I went to meet her in Ahmedabad at SEWA headquarters, and she said, “If you are ready to work shoulder-to-shoulder with informal workers, then go and finish your education and come back to join the movement.” When I finished my undergraduate studies, I returned to work at a tuberculosis (TB) clinic in Mumbai and cared for ‘pavement-dwellers’ (people living on the street) for a year. They were mostly economic migrants, and many already had TB when they came to the city, but the disease was spreading like wildfire. I had seen plenty of poverty, but that experience really opened my eyes to the way socioeconomic conditions determined health, and how vulnerable informal sector workers were. When I did my Master’s, I chose to do my field work at SEWA, then joined the organization in 1984. Forty years later I am still here. My SEWA sisters hooked me for life!

*Q: What are the main health challenges faced by women working in the informal sector in India?*

A: Lack of health literacy, information, and access to services that workers in the formal sector usually obtain. Informal workers depend on their own labour and resources for survival, and most are self-employed. SEWA grew out of the women's wing of the Textile Labour Association (TLA), a labour union founded by Mahatma Gandhi in 1918; but one of the biggest challenges faced by informal workers is that they are not organized. That is why one of the first tasks undertaken by Elaben was to register a union and then support the setting-up of cooperatives across a range of economic activities. We now use our collective strength – and we currently have 2.5 million members – as a movement of unions and cooperatives to ensure that livelihood and social protection like basic good quality health services actually reach women in a timely, transparent and inclusive manner.

“Members said, “Our bodies are our only assets.”

When SEWA started out, lack of access to financial services was also a big challenge. Many of our members are micro-entrepreneurs, such as street vendors or artisans, but the banks would not lend to them 50 years ago. They claimed they were “unbankable” because they did not believe that poor women could save and return money that they borrowed. It has since been demonstrated by the women-led, worldwide micro-finance movement that this is completely unjustified, and I’d like to point out that the repayment rate for loans at SEWA Bank, our cooperative bank, is about 97%! However, there have been some issues, notably those related to women’s health. A study done by SEWA Bank in the early 1970s revealed that the number one reason for women not paying back their loans was sickness, either their own or that of a family member. Shockingly, 20 of the 500 surveyed women had died, and 15 of those deaths were due to childbirth. So, once again, the interrelationships between social determinants of health were brought to the fore – poverty impacting health, and poor health leading to or perpetuating poverty. To overcome the financing challenges women in the informal sector faced, we set up the SEWA Bank in 1974. The staff and board members were drawn from among SEWA members, and the capital also came entirely from the members who are the shareholders. It was they who set the loan rates. By doing this we were able to improve SEWA members' financial well-being.

*Q: How did SEWA go about addressing the problem of health service access?*

A: By setting up a community-based primary health care programme for SEWA members. India was a signatory to the 1978 Alma-Ata Declaration on primary health care, but when I joined SEWA in 1984 we, as a nation, were falling woefully short in terms of investing in primary health care. Elaben broke the challenge down into three inter-connected issues. The first was access to health care itself. Members said, “Our bodies are our only assets; as long as we have eyes to see, and our hands and feet work, we can feed our children. So help us stay healthy.” The second was the cost of health care. Members said that they worked hard but most of their hard-earned money went on medicine and doctors’ bills, and to pay back health-care debts. The third was exposure to health risks related to their work which was often hazardous.

On the health service provision front, we started training SEWA members to be health workers and educators. Our view was that, given the resource constraints we faced, the only way we were going to make progress on health was through collective action and through active citizen engagement. This was particularly important for the most vulnerable people who are typically overlooked, if not actively excluded, by the public and private health care systems. You have to remember our members are often from the most disadvantaged castes (social groups) and communities and minorities. So, we focused on training these women as health workers and health educators.

Education is of paramount importance because one of the biggest barriers to health service access is lack of information. Women don't know what government health schemes and entitlements, including food, are available to them or how to access them. We support them in navigating the system, particularly on the digital side, and have set up SEWA Shakti Kendras – decentralized empowerment centres located near where women live and work. The centres are not clinics, but rather information hubs offering structured training on health, nutrition, and health literacy, largely catering to women and adolescents. They also provide the information and support needed to ensure access to health services and entitlements.

In 1990 we set up India’s first health cooperative, Lok Swasthya Mandli or People’s Health Cooperative, a sister organization of SEWA which provides preventive, promotive and curative health services through a cadre of grassroots-level female health workers. They take health care to the workers’ doorsteps, providing basic health information, primary health care services and referrals to higher levels of care. We also focus on immunization and action on nutrition, and address broader social determinants of health like water and sanitation.

To ensure representation and accountability, we work closely with local public health authorities to revitalize and strengthen community mechanisms. Every village and urban settlement should have an active health committee of local women and men tasked with ensuring that clean water and sanitation are available under the National Health Mission of the Government of India. We work to support capacity-building of these committees and ensure that female leaders serve on them. The SEWA Shakti Kendra hubs I mentioned are also important here, acting as hubs to link with the government's public health services. These centres are located across Gujarat state and in another 10 states across India.

“We the citizens have to act and act together.”

*Q: You mentioned the difficulties your members had in paying their medical bills. What have you done to address the problem of out-of-pocket payments?*

A: In the first instance, we went to the insurance companies, rather like we went to the banks to get financing in the 1970s, and we received a similar response. They said, “Informal workers are a bad risk, they get sick and die too much.” So, we developed VimoSEWA, which was India’s first all-women insurance cooperative with shareholders from five states. Female informal workers are the policy-holders, owners, users and managers of VimoSewa, and they pay an annual premium of around 100 rupees (1.20 United States dollars) upwards to access insurance. We cannot be a fully-fledged insurer because regulations require one billion rupees in capital which we do not have, but we can still do a lot, and now mainly work with mainstream insurance companies who have changed their views about us. We co-create products, and our members can insure their husbands and children as well.

I’d also like to point out that the government drew on our experience with VimoSEWA when setting up Rashtriya Swasthya Bima Yojana (a government-run health insurance programme for the Indian poor), and later the newer health insurance scheme, Pradhan Mantri Jan Arogya Yojana. Similarly, the government took inspiration from our chain of low-cost 24/7 pharmacies providing generic drugs and some branded drugs at low cost in setting up their Jan Aushadhi Pariyojana scheme, which was launched to provide quality generic medicines at affordable prices.

*Q: What is next on SEWA’s agenda?*

A: We've been spearheading a national campaign to raise demand for universal health coverage (UHC). The government has expressed its commitment to UHC and the Ayushman Bharat programme is a start, but government spending on health remains low at around 2.1% of the Gross Domestic Product (GDP), and out-of-pocket expenses make up 62% of India's total health-care expenditure. So, much more needs to be done and it is vital that the government increase its investment. We also believe that there are enormous opportunities to advance the public health agenda by coming together, building unity and solidarity. Decentralized primary health care plans made with people locally are the next step. The government obviously has a major responsibility and stewardship role, but in a country of 1.4 billion people, we the citizens also have to act and act together.

